# Food security status and associated factors among cash-beneficiary and non-beneficiary women in the urban productive safety net program in Gondar City, Northwest Ethiopia

**DOI:** 10.1186/s40795-026-01437-7

**Published:** 2026-07-28

**Authors:** Yosef Gete Mekonone, Netsanet Worku Mengistu, Lemlem Daniel Baffa

**Affiliations:** Department of Human Nutrition, Institute of Public Health, College of Medicine and Health Science, University of Gondar, Gondar, Ethiopia

**Keywords:** Food security, Food Consumption Score, UPSNP, Cash beneficiary, Non-cash beneficiary, Gondar, Ethiopia

## Abstract

**Background:**

In Ethiopia and other low- and middle-income nations, food insecurity is still a serious public health issue, especially for women who are of reproductive age. Food security and dietary diversity are commonly measured using the Food Consumption Score (FCS). There is, however, no data comparing the food security situation of cash users and non-beneficiaries of the Urban Productive Safety Net Program (UPSNP). The purpose of this study was to evaluate these groups’ levels of food security and related variables in Gondar City, Northwest Ethiopia.

**Method:**

From May to July 2025, 720 women participated in a community-based comparative cross-sectional study (364 beneficiaries and 356 non-beneficiaries). Simple random sampling was employed after a multistage sampling process. A standardized questionnaire based on World Food Programme guidelines was used to gather data. The Food Consumption Score was used to quantify food security status, which was then divided into three categories: poor, borderline, and acceptable. To find related factors, ordinal logistic regression was employed. 95% confidence intervals were used to determine statistical significance at *p* < 0.05.

**Results:**

Among cash-beneficiary women, 50.0% had acceptable Food Consumption Scores (FCS), compared with 20.8% among non-beneficiaries. Cash-beneficiary women were significantly more likely to achieve a higher FCS than non-beneficiaries. Factors significantly associated with FCS included beneficiary status, age, dietary habits, and nutritional knowledge. Women with good dietary habits had higher odds of achieving acceptable FCS, whereas older age groups and women with good nutritional knowledge had lower odds of higher FCS.

**Conclusion:**

Food consumption among women remained suboptimal, particularly among non-beneficiaries. Cash assistance was associated with improved food consumption outcomes. Strengthening nutrition-sensitive social protection programs, promoting healthy dietary practices, and addressing socioeconomic barriers may improve food consumption among vulnerable urban women.

## Background

Food insecurity continues to be a significant global public health concern, impacting billions of individuals globally [1]. The burden is especially heavy in low- and middle-income nations, particularly in Sub-Saharan Africa, where conflicts, economic instability, and climate shocks continue to jeopardize nutritional quality and food access [2]. Food security affects approximately 258 million individuals worldwide and is defined as consistent access to sufficient, safe, and nutritious food necessary for an active and healthy life. One in three persons in Sub-Saharan Africa experiences moderate to severe food insecurity, with children in developing countries being especially vulnerable [3, 4].

According to the Ethiopian government, 15.8 million people will be hungry and require food assistance by 2024. This includes 7.2 million individuals who need immediate aid due to severe food insecurity [5]. Of Ethiopia’s nearly 33 million people who are food insecure and chronically undernourished, 25% require urgent help [6]. Women have specific nutritional requirements throughout their lives [7]. However, poor dietary intake remains the leading cause of illness and death among women and children [8]. Eating is a repetitive activity influenced by a multitude of factors—such as time of day, physiological needs, sensory stimulation, and social settings—that vary throughout the day. These factors affect not only the frequency of eating but also the types of food consumed, and the overall dietary patterns observed within households. The Food Consumption Score (FCS) is a food security indicator that measures the diversity and frequency of foods consumed over the previous seven days at the household level [9, 10]. The World Food Programme (WFP) currently recommends an acceptable FCS threshold of 90%; however, the global acceptable FCS has significantly declined, leading to insufficient food consumption and posing a threat to public health [11, 12]. Food insecurity remains a significant global challenge. Recent estimates indicate that between 713 and 757 million people experienced hunger in 2023, while approximately 2.33 billion people, representing nearly one-third of the world’s population, faced moderate or severe food insecurity [13]. These findings demonstrate the continuing global burden of inadequate access to sufficient and nutritious food and its implications for health and well-being. The highest rates of hunger are found in Sub-Saharan African (SSA) countries, where one in four people suffers from malnutrition [14]. A 2024 WFP evaluation found that 74% of Ethiopia’s population has appropriate national FCS coverage [5].

Vulnerable populations are more likely to have poor dietary intake due to food insecurity, household expenses and size, social instability, and rising food prices [15, 16]. With a significant portion of the population experiencing food insecurity.

Ethiopia has implemented several initiatives to tackle this issue, most notably the Productive Safety Net Program (PSNP). Launched in 2016, it is one of the largest social protection programs in Africa, aimed at improving food security and resilience among at-risk populations [17]. Ethiopia continues to face severe food insecurity due to the effects of drought, displacement, climate change, poverty, inflation, conflict, lack of access to safe drinking water, poor sanitation, and inadequate food intake, which particularly affects vulnerable women [18].

Undernutrition is the greatest threat to public health, according to the World Health Organization (WHO) [19]. Maternal malnutrition in Ethiopia results from highly sensitive intra-household food distribution practices [20]. Recent Demographic and Health Surveys (DHS) indicate that Ethiopia has a higher prevalence of maternal undernutrition compared to many other African countries. In sub-Saharan Africa, the proportion of undernourished mothers ranges from 7% to 37% [21]. Specifically, data from the Ethiopian Demographic and Health Surveys (EDHS) conducted in 2000, 2005, and 2011 show that the prevalence of undernutrition among Ethiopian women was 30.5%, 26.9%, and 27%, respectively [22].

Since 2016, Ethiopia’s Productive Safety Net Program (PSNP) has aimed to reduce chronic food insecurity by providing regular food support to vulnerable populations. However, despite improvements in household resilience, food insecurity and undernutrition—particularly among women and children in the Amhara Region—remain persistent and serious challenges [23]. Although food insecurity significantly impacts women’s nutrition, there is limited evidence comparing food consumption between cash-beneficiary and non-beneficiary women in urban Ethiopia. Therefore, this study aimed to assess the food security status and identify associated factors among cash-beneficiary and non-cash-beneficiary women in Gondar City, Ethiopia.

## Methods and materials

### Study area, design and period

From May to July 2025, a community-based comparative cross-sectional study was conducted in Gondar City, Northwest Ethiopia, comparing women who received monetary benefits with those who did not. Gondar is located in the Amhara National Regional State (ANRS), 180 km from Bahir Dar, the regional capital, and approximately 750 km northwest of Addis Ababa. The city is divided into six sub-cities and twenty-one kebeles and is situated at an elevation of 12°8′ N and 37°28′ E. According to 2025 municipal government data, Gondar has a population of 443,156, comprising 217,107 men (49%) and 226,054 women (51%). The city has 32 private clinics, 14 health posts, and 8 government health centers. The Urban Productive Safety Net Program (UPSNP), established in 2016 to support the urban poor—particularly women and the elderly—benefits a significant proportion of Gondar households. Beneficiaries are identified through community-based targeting based on labor capacity and vulnerability. Labor-constrained individuals receive unconditional cash transfers, while labor-capable beneficiaries participate in public works projects in exchange for payments. In Gondar City, 6,443 women were enrolled as cash beneficiaries, and 7,654 women were non-beneficiaries. Monthly payments are disbursed through local banks.

### Population

The source population comprised all women participating in the Urban Productive Safety Net Program (UPSNP) in Gondar City, including both cash-beneficiary and non-beneficiary women. The study population consisted of women of reproductive age residing in specific sub-cities of Gondar City who were enrolled in the UPSNP program, encompassing both current beneficiaries receiving financial aid and those registered to receive assistance in the near future. Women were eligible for inclusion if they had lived in the selected sub-cities for more than six months. Additionally, women who prepared or consumed meals at home during the seven days prior to data collection qualified for participation. Conversely, women experiencing severe physical or mental health issues that impaired their ability to participate in the interview were excluded from the study.

### Sample size and sampling procedure

The sample size was determined using Epi Info version 7 for a comparative cross-sectional study assuming a 95% confidence level, 80% power, and a 1:1 ratio of beneficiaries to non-beneficiaries. Based on previous evidence and after applying a design effect of 2 and a 10% allowance for non-response, participants. The acceptable(FCS) of 73.9% among non-beneficiary households, obtained from previous studies [24]. was used to calculate the sample size. As no prior study was conducted among cash-beneficiary households, the proportion of acceptable food consumption was assumed to decrease by 15% compared to non-cash beneficiary households, resulting in a prevalence of 58.9%.

$$\frac{\mathrm n1=\left\{\left[{\mathrm Z}_1-\mathrm\alpha/2\times\sqrt{}\left(2\times\overline{\mathrm p}\times\left(1-\overline{\mathrm p}\right)\right)+{\mathrm Z}_1-\mathrm\beta\times\sqrt{}\left({\mathrm p}_1\left(1-\mathrm p1\right)+{\mathrm p}_2\right)+{\mathrm p}_2\left(1-{\mathrm p}_2\right)\right]^2\right\}}{\left(\mathrm p1-\mathrm p2\right)^2}$$

*n*_*2*_*=*$\:r\times\:n1$, n = Sample size for cash beneficiary, α/_2_=level of significance,

Zα/_2_ = standard distribution at 95% CI. $\:\beta\:$ =80% Standard value for power.

r= Ratio of exposed to unexposed, 1:1, so *r* = 1.

p_1_= Expected prevalence of non- beneficiary p_2_= Expected prevalence of beneficiary based on the assumption the estimated sample size (no) is 336.the final sample size was 740.

The sample size for the second objective was determined using a significant factor associated with Food Consumption Score (FCS) identified in a previous study conducted in Jimma Town, Southwest Ethiopia. Using Epi Info version 7, a design effect of 2, and a 10% non-response rate, the final sample size was 563 participants [25].

A two-stage cluster sampling method was employed. In the first stage, sub-cities were treated as clusters, and 30% of the total sub-cities were selected using simple random sampling (lottery method). Consequently, two sub-cities, Arada and Maraki, were included in the study. In the second stage, women within the selected kebeles were categorized as cash beneficiaries and non-cash beneficiaries. Proportional allocation was applied based on the population size of each group in the chosen sub-cities. Specifically, in Arada, there were 230 public work beneficiaries and 121 direct cash beneficiaries, as well as 267 public work and 140 non-cash beneficiaries. In Maraki, 598 public work and 245 direct cash beneficiaries, along with 517 public work and 211 non-cash beneficiaries, were identified. Finally, study participants were selected through simple random sampling from each stratum.

### Variable of the study

#### Dependent variable

Food consumption scores: (Poor, Border line, Acceptable)

#### Independent variable

Sociodemographic factors of respondents: mother age, education, employment status, ethnicity, occupations of both the woman and her husband, presence of any disabled household member. religion, decision maker of the household, family size, household expenditure profile, wealth index.

Personal factors: include the woman’s attitude, knowledge, media exposure.

Dietary factor: Diet and Feeding Pattern, meal/day, meal skip habit of Eating snack.

### Operational definition

#### Food consumption score

Food Consumption Score was assessed according to the World Food Programme (WFP) guidelines. It is a composite indicator that reflects dietary diversity, food consumption frequency, and the relative nutritional importance of different food groups. Information on the consumption frequency of eight food groups during the seven days preceding the survey was collected. Consumption frequencies for each food group were summed and truncated at a maximum of seven days, then multiplied by their respective WFP weights. The weighted scores were summed to obtain the household Food Consumption Score. Based on standard WFP cut-off points, households were classified as having poor (< 21), borderline [21–35], or acceptable (> 35) food consumption scores [10].

#### A food group

Foods that have similar nutritional content and are commonly consumed for similar dietary purposes. In this study, foods are grouped into Eight main categories [26].

#### A food item

An individual, specific food consumed by study participants that falls within one of the predefined food groups [26].

Consumption frequency: is not the number of times a food item is consumed, but rather the number of days it was consumed throughout a seven-day period [27].

Cash-beneficiary: women who receive financial assistance through a cash transfer program that can be used for livelihood services [28].

Non-beneficiary: do not receive direct cash transfers from any social welfare programs or interventions [29].

Attitude: was measured using attitude-related questions. Respondents scoring at or above the mean score were considered to have a favorable attitude, whereas those scoring below the mean were considered to have an unfavorable attitude [30].

Knowledge: was assessed asking ten nutrition-related questions. Respondents scoring at or above the mean score were classified as having good nutrition knowledge, while those scoring below the mean were classified as having poor nutrition knowledge [31].

Media Exposure: characterized by the frequency at which women engaged with various forms of media, such as radio, television, newspapers, magazines, and social media platforms Facebook, Telegram, WhatsApp. Women who indicated that they accessed at least one of these media sources at least once a week were deemed to have media exposure, whereas those who accessed these sources less than once a week or not at all were classified as having no media exposure [32].

#### Wealth index

The household wealth index was generated using Principal Component Analysis (PCA) based on household assets, housing characteristics, and access to services. Households were categorized into poor, middle, and rich wealth groups [16].

Special Needs Household Member: a special needs member if at least one member had a physical, sensory, intellectual, or other disability requiring additional care and support [33].

Cash utilization: refers to the extent to which cash assistance received through the UPSNP was appropriately used for intended household needs, including food and essential services. [28].

#### Program impact

Program impact was assessed based on participants' perceptions of the contribution of UPSNP cash assistance to improving household well-being and food access [34].

#### Family conflict due to cash assistance

Refers to any reported disagreement, tension, or problem within the household that the respondent attributes to the receipt or use of cash assistance [35].

#### Household decision-making

Participants were inquired about who predominantly makes decisions related to household issues, such as food purchases, healthcare usage, and financial spending. The responses were classified into the following categories: respondent alone, husband/partner alone, and joint decision-making (respondent and husband/partner together) [36].

Dietary habit: was assessed using dietary practice-related questions. Participants scoring at or above the mean score were categorized as having good dietary habits, while those scoring below the mean were categorized as having poor dietary habits. The habit of meal skipping was examined by asking respondents if they had skipped any regular meals in the last seven days [37].

Eating Snack: was measured by asking respondents whether they consumed any food between main meals in the past 24 hours [38].

#### Family size

Refers to the total number of individuals living in the same household and sharing meals from the same pot. For this study, family size was categorized as small (≤ 3 members), medium (4–6 members), and large (≥ 7 members) [39].

### Data collection tools and procedure

The data collection process involved using structured questionnaires to gather a comprehensive set of information. The WFP’s standard Food Consumption Score (FCS) framework served as the basis for the questionnaire [40]. Before the interview, study participants were asked to list every food and drink they had consumed in the previous seven days to assess their FCS. Using the Ethiopian Food Composition Table [41]. Eight distinct food categories were established based on indigenous foods. This table provides standard weight values aligned with the eight food categories recognized by the World Food Programme and includes all relevant local foods consumed across different regions of the country. Injera, a staple dish, is primarily made from teff; however, it is occasionally prepared with other grains such as sorghum, maize, barley, and wheat. For example, when participants reported having eaten injera, the corresponding weight value was recorded. Condiments are generally consumed in minimal quantities, often solely for flavor enhancement, and are not considered to significantly impact the overall diet; therefore, they hold no weight in the primary food categories. In fact, there are recommendations to structure the questionnaire to include condiments during data collection. This study does not employ weight thresholds to distinguish between a food’s role as a condiment versus its role as a main dish when collecting data from interviewees. Nevertheless, we typically isolated commonly used condiments and endeavored to adhere to the general standard questionnaire. A range of factors was assessed, including household livelihoods, expenditures, cash support systems, food consumption patterns, and sociodemographic characteristics. The eight food categories that participants were queried about to determine their food consumption score were a vital component of the research. To ensure both accuracy and efficiency, in-person interviews were conducted using Kobo Toolbox, a mobile data collection platform.

### Data quality assurance

To ensure data quality, an intensive four-day training program was conducted for data collectors and supervisors, focusing on the study’s objectives, interviewing techniques, and proper questionnaire administration. Before actual data collection, role-playing exercises were implemented to familiarize the team with the tool and enhance consistency in interviewing practices. All data collectors and supervisors held a Bachelor of Science degree in health-related disciplines. Daily supervision was provided by field supervisors and the principal investigator, who reviewed each completed questionnaire for completeness, consistency, and accuracy, offering immediate feedback when necessary. The questionnaire was initially drafted in English and then translated into Amharic to ensure clarity and cultural relevance. It was subsequently back-translated into English by independent language experts to verify consistency across versions. A pretest was conducted on 5% of the total sample size in a comparable setting outside the study area, resulting in necessary adjustments based on the findings, including clarifying ambiguous questions and refining wording. To minimize recall bias, a brief recall period of seven days was used for dietary assessment (Food Consumption Score), and standardized probing questions were employed during interviews to help participants accurately recall their food consumption.

### Data processing and analysis

The collected data were imported into EpiData Version 3.1 for cleaning after being exported from the Kobo tool used for coding. Following the cleaning process, the dataset was exported to STATA Version 17.0 for analysis. Sociodemographic characteristics and other independent variables were summarized using descriptive statistics, including mean, standard deviation, median, frequency, percentage, and percentiles. Ordinal logistic regression was employed to identify characteristics significantly associated with the dependent variable, the Food consumption score (FCS), which was reclassified into three categories: poor, borderline, and acceptable. The essential assumptions of binary logistic regression were evaluated before fitting the model. The Variance Inflation Factor (VIF) was used to assess multicollinearity among independent variables. A conservative cutoff value of VIF < 5 was applied to ensure minimal collinearity among predictors, although values below 10 are generally considered acceptable. To address potential confounders, bivariable binary logistic regression was initially conducted, and variables with a p-value < 0.25 were included in the multivariable model. Statistical significance in the multivariable analysis was determined at *p* < 0.05, with both Crude Odds Ratios (COR) and Adjusted Odds Ratios (AOR) reported alongside 95% Confidence Intervals (CI). Principal Component Analysis (PCA) was used to develop the wealth index. The final results were presented using tables, graphs, and numerical summaries.

## Results

### Sociodemographic and economic factors of respondent

A total of 740 individuals were initially selected for the study. Of these, 720 completed the interview process, yielding a response rate of 97.3%. Twenty participants either did not respond or were excluded due to incomplete data. Within the final sample, 364 (50.6%) were cash beneficiaries, while 356 (49.4%) were classified as non-cash beneficiaries. The beneficiary group had a higher proportion of individuals aged 35–44 (38.7%), whereas non-beneficiaries were primarily younger, specifically in the 25–34 age range (46.6%). Educational attainment was generally low, with over 80% of both groups having only primary education or less. A larger percentage of beneficiaries were categorized as poor (48.9%), compared to a greater proportion of non-beneficiaries who were classified as rich (40.4%). More than half of the respondents (53.2%) were married. The overwhelming majority of respondents in both groups (over 98%) identified as Amhara ethnicity. These findings are summarized in Table 1.

**Table 1:** Sociodemographic characteristics of beneficiaries (*n* = 364) and non-beneficiaries (*n* = 356), Gondar City, Ethiopia, 2025. Values are presented as n (% column)

Variable	Category	Frequency (%)		Percentage (*n* = 720)
		Beneficiary (*n* = 364)	Non-beneficiary (*n* = 356)	
Age group	18–24	22 (6.0%)	50 (14.0%)	10.0
	25–34	110 (30.2%)	166 (46.6%)	38.3
	35–44	141 (38.7%)	108 (30.3%)	34.5
	45–49	63 (17.3%)	60 (16.9%)	17.1
Educational level of respondent	Can not read and write	108 (29.7%)	150 (42.1%)	35.8
	Primary education	189 (51.9%)	154 (43.3%)	47.6
	Secondary education	60 (16.5%)	41 (11.5%)	14.5
	Higher education	11 (3.0%)	7 (2.0%)	2.5
Religion	Muslim	63 (17.3%)	66 (18.5%)	17.9
	Orthodox	286 (78.6%)	266 (74.7%)	76.6
	Protestant	24 (6.5%)	15 (4.2%)	5.4
Ethnicity	Amhara	363 (99.7%)	349 (98.0%)	98.8
	Tigrayan	1 (0.3%)	7 (2.0%)	1.1
Occupation of respondent	Daily laborer	70 (19.2%)	110 (30.9%)	25.0
	Employers	33 (9.1%)	39 (11.0%)	10.0
	Housewife	112 (30.8%)	80 (22.5%)	26.6
	private workers	149 (40.9%)	127 (35.7%)	38.3
Family size	Small size	117 (32.1%)	194 (54.5%)	43.2
	Medium size	217 (59.6%)	156 (43.8%)	51.8
	Large size	30 (8.2%)	6 (1.7%)	5.0
Household decision making	Without mother involvement	24 (6.6%)	38 (10.7%)	8.6
	With mother involvement	340 (93.4%)	318 (89.3%)	91.4
Wealth index	Poor	132 (37.0%)	178 (48.9%)	43.5
	Middle	80 (22.5%)	88 (24.2%)	23.3
	Rich	144 (40.4%)	98 (26.9%)	33.6

### Knowledge, attitude, and dietary habits of respondents

A significant proportion of beneficiaries (72.3%) demonstrated a strong understanding of the program, compared to only 43.8% of non-beneficiaries who exhibited similar levels of awareness. Attitudes toward food consumption were predominantly positive and comparable across both groups, with 77.2% of beneficiaries and 78.7% of non-beneficiaries expressing favorable attitudes. Beneficiaries also reported better dietary habits, with 61.0% adhering to healthy dietary patterns, compared to 43.0% of non-beneficiaries. Regarding daily meal frequency, 62.4% of beneficiaries consumed meals more than three times a day, whereas 82.0% of non-beneficiaries ate fewer than three meals daily. Snacking behaviors were minimal in both groups, though slightly more common among beneficiaries (15.9%) than non-beneficiaries (7.9%), with the majority in both groups refraining from snack consumption (84.1% of beneficiaries and 92.1% of non-beneficiaries). Notably, media exposure was higher among non-beneficiaries, with 85.4% reporting access compared to 67.6% of beneficiaries, as shown in Table 2.

**Table 2:** Knowledge, attitude towards FCS and dietary habits of study respondents in Gondar city, Ethiopia 2025 (*n* = 720)

Variable	Category	Frequency (%)		Percentage (%) *n* = 720
		Beneficiary (*n* = 364)	Non-beneficiary (*n* = 356)	
Knowledge	Good	263	156	58.2
	Poor	101	200	41.8
Attitude	Favorable attitude	281	280	77.9
	Unfavorable attitude	83	76	22.1
Dietary Habits	Good habit	222	153	52.1
	poor habit	142	203	47.9
Media exposure	Yes	246	304	76.4
	No	118	52	23.6
Daily Feeding pattern	Less than 3 times	137 (37.6%)	292 (82.0%)	59.6
	More than 3 times	227 (62.4%)	64 (18%)	40.4
Eating snack	No	306 (84.1%)	328 (92.1%)	88.5
	Yes	58 (15.9%)	28 (7.9%)	11.9

### Mother food group consumption in the past seven days

The intake of fruit, meat, and milk was low for both groups, each constituting less than 10% of their diet. However, a significant disparity was observed in vegetable consumption, with non-beneficiaries consuming more (61.2%) than beneficiaries (47.8%), as shown in Table 3.

**Table 3:** Household food group consumption in the past seven days in Gondar city, Ethiopia 2025 (*n* = 720)

Type of Food	Feeding Category	Frequency (%)	
		Beneficiary (*n* = 364)	Non-Beneficiary (*n* = 356)
Main staples	No	2 (0.6%)	2 (0.6%)
	Yes	362 (99.4%)	354 (99.4%)
Pulse	No	5 (1.4%)	0 (0.0%)
	Yes	359 (98.6%)	356 (10%)
Fruits	No	334 (91.8%)	330 (92.7%)
	Yes	30 (8.2%)	26 (7.3%)
Vegetable	No	190 (52.5%)	138 (38.7%)
	Yes	174 (47.8%)	218 (61.2%)
Meat	No	330 (90.7%)	336 (94.4%)
	Yes	340 (8.2%)	20 (5.6%)
Milk	No	334 (91.8%)	343 (96.3%)
	Yes	30 (8.2%)	13 (3.6%)
Oil	No	5 (1.4%)	0 (0.0%)
	Yes	359 (98.6%)	356 (100%)
Sweets	No	20 (5.5%)	18 (4.9%)
	Yes	344 (94.5%)	338 (94.9%)

### Mother FCS with food group consumption in the past seven days

The consumption of staple foods, pulses, oils, and sweets was nearly universal across all Food Consumption Score (FCS) categories. In contrast, the intake of nutrient-rich foods such as fruits, meat, and milk remained consistently low, with fewer than 5% of households reporting consumption in each FCS group. The distribution of households among poor, borderline, and acceptable FCS categories was similar for the most frequently consumed food groups. However, households classified within the acceptable FCS category showed a slightly higher proportion of meat consumption compared to the other categories. Data collection took place during a non-fasting period, minimizing the potential influence of religious dietary restrictions on food consumption patterns. Therefore, the observed low intake of animal-source and micronutrient-dense foods is unlikely to be due to fasting practices and more likely reflects actual household dietary habits (see Table 4).

**Table 4:** Food Consumption Score of study participants in Gondar city, Ethiopia, 2025 (*n* = 720)

Food groups		Food consumption score Frequency (%)		
		Poor	Borderline	Acceptable
Main staples(cereals)	Yes	144 (20%)	316 (43.9%)	256 (35.5%)
	No	3 (0.4%)	1 (0.1%)	0 (0.0%)
Pulses	Yes	145 (20.1%)	314 (43.6%)	256 (35.6%)
	No	2 (0.3%)	3 (0.4%)	0(0.0%)
Fruits	Yes	9 (1.2%)	20 (2.8%)	27 (3.8%)
	No	138 (19.2%)	297 (41.25%)	229 (31.8%)
Vegetables	Yes	80 (11.1%)	213 (29.6%)	157 (21.8%)
	No	67 (9.3%)	104 (14.4%)	97 (13.5%)
Meat	Yes	10 (1.4%)	11 (1.5%)	33 (4.6%)
	No	137 (19.0%)	306 (42.5%)	223 (30.9%)
Milk	Yes	8 (1.1%)	19 (2.6%)	16 (2.2%)
	No	139 (19.3%)	298 (41.4%)	240 (33.3%)
Oil	Yes	147 (20.4%)	313 (43.5%)	255 (35.4%)
	No	0 (0.0%)	4 (0.6%)	1 (0.1%)
Sweets	Yes	133 (18.5%)	302 (41.9%)	245 (34.0%)
	No	14 (1.9%)	15 (2.1%)	11 (1.5%)

### Women household expenditure patterns

The examination of typical monthly household spending indicated that food constituted the largest expense, averaging 1,475.85 ETB. Significant additional costs included cooking fuel (147.22 ETB), water (126.81 ETB), and hygiene items (111.13 ETB). Conversely, the average expenditure on healthcare was remarkably low (8.56 ETB), as shown in Table 5.

**Table 5:** Expenditure profile for the study participants in Gondar city, Ethiopia 2025. (*n* = 720)

Household expenditure	Mean ± Std. Deviation
Food	1475.8 ± 538.7
Water	126.81 ± 114.1
Hygienic products	111.1 ± 69.4
Cooking fuel	147.2 ± 128.6
Medical cost	8.5 ± 52.6
Clothes	108.4 ± 334.00
Communication	63.5 ± 59.1

### Food consumption score by beneficiary status

A chi-square test of independence indicated a statistically significant relationship between beneficiary status and FCS category, χ² (2, *N* = 720) = 68.64, *p* < 0.001.

Among the beneficiary group (*n* = 364), 64 households (17.6%) were categorized as having a poor food consumption score. Additionally, 118 households (32.4%) fell into the borderline category, while 182 households (50.0%) achieved an acceptable food consumption score. This indicates that half of the beneficiary households met the minimum dietary diversity and frequency standards considered acceptable.

In contrast, women from non-beneficiary households (*n* = 356) exhibited less favorable outcomes. Specifically, 83 households (23.3%) were classified as having poor food consumption scores, while a significant majority of 199 households (55.9%) fell into the borderline category. Only 74 households, representing 20.8% of the non-beneficiaries, maintained acceptable food consumption levels.

**Table:** 

FCS category	Non-beneficiaries (*n* = 356) % (95% CI)	Beneficiaries (*n* = 364) % (95% CI)
Poor	23.3% (19.2–27.9)	17.6% (13.9–21.8)
Middle	55.9% (50.7–61.0)	32.4% (27.8–37.4)
Acceptable	20.8% (16.8–25.2)	50.0% (44.9–55.1)

### Food consumption score among beneficiaries

**Fig. 1:**
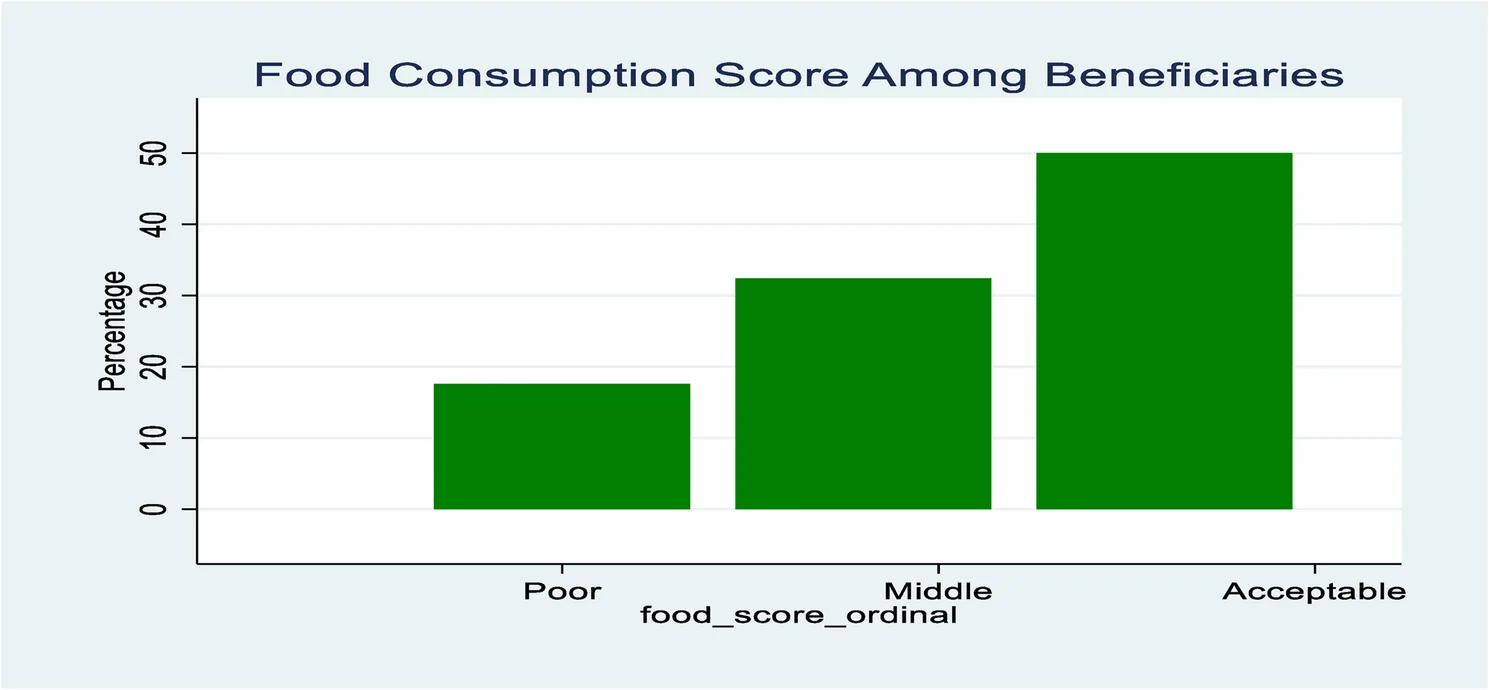
Food consumption score among UPSNP cash-beneficiary women in Gondar City, Northwest Ethiopia, 2025 (*n* = 364)

Among the 364 cash beneficiary participants, 50% (*n* = 182) exhibited an acceptable food consumption score, indicating a relatively favorable dietary intake in terms of both frequency and diversity. Conversely, 32.42% (*n* = 118) were classified within the middle food consumption category, suggesting a moderate level of food access and diversity. In contrast, 17.58% (*n* = 64) of the beneficiaries were identified as having a poor food consumption score, highlighting significant dietary deficiencies and a potential risk of food insecurity. These results are presented in Fig. 1.

### Food consumption score for non-beneficiaries

**Fig. 2:**
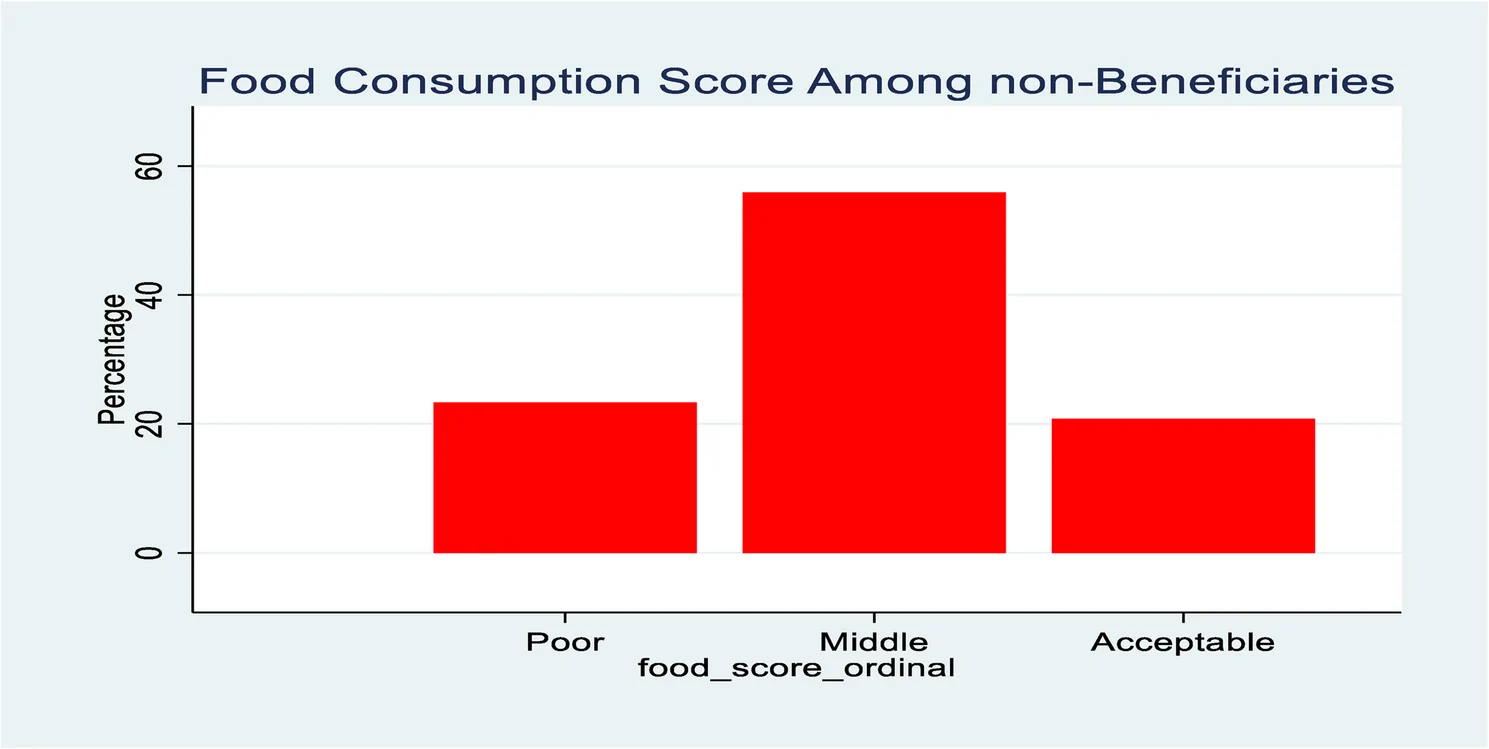
Food consumption score among UPSNP cash non-beneficiary women in Gondar City, Northwest Ethiopia, 2025 (*n* = 356)

The analysis of the Food Consumption Score among non-beneficiaries reveals that a significant portion falls within the “middle” category, with 199 individuals representing 55.90% of the total sample (*N* = 356). Those classified as having a “poor” food consumption score number 83, accounting for 23.31% of the sample. In contrast, the “acceptable” category includes 74 individuals, comprising 20.79%. While more than half of the non-beneficiaries exhibit a moderate food consumption, nearly a quarter are classified as having poor food consumption. The cumulative distribution shows that 79.21% of non-beneficiaries fall within the combined poor and middle categories, as illustrated in Fig. 2.

### Factors associated with Food consumption score

Variables with *p* ≤ 0.25 in the bivariate analysis were included in the multivariable ordinal logistic regression to avoid excluding potential confounders. Multicollinearity was ruled out (*r* < 0.5, VIF < 5, tolerance > 0.2). The model demonstrated a good fit across all samples (LR χ² = 39.04, *p* = 0.0004; 36.21, *p* = 0.0145; 30.44, *p* = 0.0066), with Pseudo R² values of 25.7%, 14.8%, and 16.4%. The Brant and likelihood ratio tests indicated no violation of the proportional odds assumption (*p* > 0.05), and link tests confirmed the correct specification of the model. In summary, the ordinal logistic regression models were appropriate and well-fitted.

### Factors associated with food consumption score for the whole respondents

Ordinal logistic regression analysis indicated that age, beneficiary status, and dietary habits were significantly associated with (FCS). Women aged 35–44 and 45–49 exhibited lower odds of achieving a higher FCS (Adjusted Odds Ratios [AOR] = 0.53 and 0.39, respectively) compared to those aged 18–24. Individuals receiving cash benefits were 2.72 times more likely to attain an acceptable FCS (AOR = 2.72; 95% Confidence Interval [CI]: 1.87–3.95). Furthermore, maintaining good dietary practices was associated with increased odds of a higher FCS (AOR = 1.52; 95% CI: 1.12–2.06), whereas possessing good nutrition knowledge was unexpectedly associated with a 30% reduction in the odds (AOR = 0.70; 95% CI: 0.50–0.98) Table 6).

**Table 6:** Multivariable ordinal logistic regression analysis of factors associated with food consumption score among all respondents in Gondar City, Ethiopia, 2025 (*n* = 720)

Variable	Category	COR (95% CI)	AOR (95% CI)	*p*-value
Beneficiary Status	Non-Beneficiary (Ref)	**1.00**	**1.00**	
	Beneficiary	**2.78 (1.83**,** 4.22)**	**2.72 (1.57**,** 3.82)**	< 0.001**
Knowledge Level	**Poor (**Ref)	**1.00**	**1.00**	
	**Good**	**0.76 (0.40**,** 0.91)**	**0.70 (0.34**,** 0.99)**	0.035**
Women’s Dietary Habit	**Poor** (Ref)	**1.00**	**1.00**	
	**Good**	**1.62 (1.07**,** 2.80)**	**1.52 (1.02**,** 2.58)**	0.043**
Age Group	**18–24** (Ref)	**1.00**	**1.00**	
	**25–34 years**	**0.52 (0.20**,** 1.34)**	**0.56 (0.21**,** 1.48)**	**0.241**
	**35–44 years**	**0.34 (0.12**,** 0.98)**	**0.53 (0.23**,** 0.79)**	0.022**
	**45–49 years**	**0.31 (0.09**,** 1.06)**	**0.36 (0.10**,** 1.27)**	**0.112**
Health Insurance	**No** (Ref)	**1.00**	**1.00**	
	**Yes**	**1.95 (1.05**,** 3.98)**	**1.62 (0.77**,** 3.41)**	**0.203**
Wealth Index	**Poor** (Ref)	**1.00**	**1.00**	
	**Middle**	**1.66 (1.13**,** 3.33)**	**1.55 (0.75**,** 3.21)**	**0.237**
	**Rich**	**1.04 (0.50**,** 2.14)**	**0.95 (0.45**,** 2.03)**	**0.902**

*: Bold values indicate statistically significant association at *p*-value < 0.05

### Factors associated with food consumption score among beneficiary women

Among households receiving cash benefits, ordinal logistic regression identified several significant predictors of the food consumption score (LR χ² = 118.71, df = 31, *p* < 0.001). Increasing age was negatively correlated with the food consumption score—women aged 25–34, 35–44, and 45–49 had 72%, 77%, and 89% lower odds, respectively, of achieving a higher food consumption score compared to those aged 18–24. Additionally, larger family size (AOR = 0.13; 95% CI: 0.06–0.28), the presence of a household member with special needs (AOR = 0.42; 95% CI: 0.23–0.74), and disputes regarding cash assistance (AOR = 0.17; 95% CI: 0.09–0.24) were associated with reduced food consumption scores. In contrast, mothers who maintained healthy dietary practices were twice as likely to attain an acceptable food consumption score (AOR = 2.01; 95% CI: 1.21–3.34), as shown in Table 7.

**Table 7:** Significant predictors of food consumption score for the beneficiary respondents in Gondar City, Ethiopia, 2025. (Multivariate ordered logistic regression, *n* = 364)

Variable	Category	COR (95% CI)	AOR (95% CI)	*p*-value
Age Group	18–24 years	1.00	1.00	
	25–34 years	0.60 (0.30, 1.22)	0.28 (0.13, 0.64)	0.002*
	35–44 years	0.47 (0.23, 0.91)	0.23 (0.10, 0.55)	0.001*
	45–49 years	0.28 (0.13, 0.59)	0.11 (0.04, 0.30)	< 0.001*
Special Need in Household	No	1.00	1.00	
	Yes	0.64 (0.52, 0.96)	0.42 (0.23, 0.74)	0.003*
Family Size	Small	1.00	1.00	
	Medium	0.65 (0.45, 0.94)	0.79 (0.49, 1.28)	0.338
	Large	0.23 (0.05, 0.19)	0.13 (0.06, 0.28)	< 0.001*
Women’s Dietary Habit	Poor	1.00	1.00	
	Good	2.60 (1.14, 2.53)	2.01 (1.21, 3.34)	0.007*
Family conflict due to assistance	No	1.00	1.00	
	Yes	0.22 (0.12, 0.46)	0.17 (0.09, 0.24)	< 0.001*
Attitude Level	Poor	1.00	1.00	
	Good	1.89 (1.43, 2.20)	1.63 (1.01, 2.63)	0.045*
Knowledge Level	Poor	1.00	1.00	
	Good	0.62 (0.36, 0.87)	0.59 (0.36, 0.95)	0.029*
Mother’s Occupation	Others	1.00	1.00	
	Employers	1.23 (0.432, 1.82)	1.10 (0.50, 2.43)	0.811
	Housewife	2.08 (1.61, 2.70)	1.50 (0.77, 2.92)	0.235
	Private Workers	1.03 (1.00, 1.20)	1.15 (0.65, 2.04)	0.621
Cash Utilization	Poor	1.00	1.00	
	Good	1.21 (1.12, 1.49)	1.10 (0.55, 2.19)	0.787
Program Impact	Low	1.00	1.00	
	Significant	0.65 (0.11, 2.66)	0.54 (0.11, 2.66)	0.453
Educational Level	Uneducated	1.00	1.00	
	Primary	1.31 (1.12, 1.54)	1.16 (0.70, 1.90)	0.568
	Secondary	1.48 (1.34, 2.01)	1.33 (0.64, 2.77)	0.449
	Higher	3.00(2.37, 6.42)	2.11(0.37, 12.00)	0.401

*: statistically significant at *p*-value < 0.05

### Factors associated with food consumption score among non-beneficiary

Among non-cash beneficiaries, ordinal logistic regression indicated that the model was significant (LR χ² = 100.18, df = 24, *p* < 0.001). Middle-wealth households had higher odds of better Food Consumption Scores (FCS) (AOR = 1.98; 95% CI: 1.19–3.85), whereas large family size (AOR = 0.04; 95% CI: 0.01–0.12) and having a member with special needs (AOR = 0.54; 95% CI: 0.31–0.94) were associated with lower FCS. Favorable nutrition attitude (AOR = 1.30; 95% CI: 0.88–1.70), good nutrition knowledge (AOR = 1.73; 95% CI: 1.05–2.86), and good dietary practices (AOR = 1.24; 95% CI: 0.21–0.96) were positively associated with higher food consumption, as shown in Table 8.

**Table 8:** Significant predictors of food consumption score for the non-beneficiary respondents in Gondar City, Ethiopia, 2025. (Multivariate ordered logistic regression, *n* = 356)

Variable	Category	COR (95% CI)	AOR (95% CI)	*p*-value
Wealth Index	Poor	1.00	1.00	
	Middle	1.30 (1.10, 1.61)	1.98 (1.15, 3.67)	0.015*
	Rich	0.85 (0.55, 1.31)	0.98 (0.59, 1.63)	0.948
Family Size	Small	1.00	1.00	
	Medium	0.65 (0.45, 0.94)	0.79 (0.49, 1.28)	0.338
	Large	0.65 (0.41, 0.93)	0.04 (0.01, 0.11)	< 0.001*
Special Need in Household	No	1.00	1.00	
	Yes	0.65 (0.42, 0.98)	0.54 (0.32, 0.98)	0.041*
Knowledge Level	poor	1.00	1.00	
	Good	1.50 (1.10, 2.05)	1.73 (1.09, 2.93)	0.032*
Attitude Level	Poor	1.00	1.00	
	Good	1.70 (1.23, 2.15)	1.30 (0.98, 2.71)	0.042*
Women’s Dietary Habit	poor	1.00	1.00	
	Good	1.61 (1.20, 2.15)	1.24 (1.02, 3.30)	0.003*
Mother’s Occupation	Other	1.00	1.00	
	Employers	1.20 (0.60, 2.40)	1.13 (0.51, 2.51)	0.770
	Housewife	1.55 (0.90, 2.65)	1.50 (0.81, 2.79)	0.195
	Private Workers	1.80 (1.05, 3.10)	1.73 (1.01, 2.95)	0.046*
Age Group	18–24 years	1.00	1.00	
	25–34 years	1.25 (0.70, 2.25)	1.39 (0.54, 3.59)	0.500
	35–44 years	1.30 (0.65, 2.60)	1.40 (0.49, 4.00)	0.530
	45–49 years	2.20 (1.10, 4.40)	2.57 (0.81, 8.17)	0.109

*: statistically significant at *p*-value < 0.05

## Discussion

Cash-beneficiary women had a significantly higher proportion of acceptable Food Consumption Scores (FCS) (50%) compared to non-beneficiary women (20.8%), and a lower prevalence of poor FCS (17.6% vs. 23.3%) (*p* < 0.001, chi-square test), highlighting the potential role of cash transfers in improving household food consumption patterns. The percentage of non-beneficiaries in the borderline group was significantly greater (55.9%) than that of beneficiaries (32.4%). These statistically significant differences indicate that women in Gondar City who participate in the Urban Productive Safety Net Program have higher food consumption scores. Initiatives are needed to sustainably improve diet quality for both populations.

The proportion of women with acceptable Food Consumption Scores observed in this study was higher than reports from several food-insecure populations in SSA where dietary quality and food security remain major challenges. Evidence from 33 Sub SSA countries showed that only 9.9% of children achieved a minimum acceptable diet, reflecting substantial nutritional vulnerabilities across the region [42].

### Food consumption score among beneficiaries in context of other studies

17.6% of the women who received cash benefits had poor food consumption scores, 32.4% had borderline scores, and 50.0% had good scores. The proportion of women with appropriate food consumption scores in this study is considerably lower than those reported in other studies. For example, studies conducted at Shegaw Motta Hospital in Northwest Ethiopia [15]. Haramaya District in Eastern Ethiopia [16] and Owo Township, Ondo State, Nigeria [43] study conducted in five woredas of East Gojjam Zone, Amhara, Ethiopia [44] Jashore district. in Polonnaruwa districts and Nuwaraeliya in Indonesia districts [45] reported acceptable food consumption scores of 81.3%, 54.4%, 56.1%, 80.3%, and 94.7%, respectively. This variation may be attributed to factors such as poor food availability, inadequate food consumption, unhealthy eating habits, differences in the study communities, and characteristics of the study participants. The lower food consumption score (FCS) in our study could also be explained by the timing of data collection; for instance, our study was conducted during the post-harvest season, whereas the Shegaw Motta study was conducted during the main harvest season [15]. Additionally, our results fall below the 74% threshold recommended by the World Food Programme (WFP) and the National Food Consumption Score Vulnerability Assessment and Mapping report on Ethiopia’s food consumption levels [46] as well as the 90% benchmark reported elsewhere [47] However, our findings are substantially higher than those from a study conducted at the Federal University of Agriculture, Abeokuta (FUNAAB), which reported an acceptable FCS of only 19.4% [48] and a study in Côte d’Ivoire during the lean season, which reported 33.6% [49]. Although both of these studies used similar research methodologies, differences in sample populations likely contributed to the disparities, possibly due to issues related to food availability and affordability. In our study, poor FCS was observed in 17.6% of participants, and borderline FCS in 32.4%. These figures are notably higher than those reported in previous research conducted in the Konso Zone in southern Ethiopia, which found 17.4% borderline and 14.1% poor FCS [10], as well as studies in Amhara (11%), Tigray (16%), Afar (6%), and Somali (17%) regions, and among women attending antenatal care at Shegaw Motta Hospital, East Gojjam Zone, Ethiopia, where poor and borderline FCS were 1.9% and 16%, respectively [14] as well as studies in Amhara (11%), Tigray (16%), Afar (6%), and Somali (17%) regions, and among women attending antenatal care at Shegaw Motta Hospital, East Gojjam Zone, Ethiopia, where poor and borderline FCS were 1.9% and 16%, respectively [15]. The discrepancies may be explained by differences in study settings and participant characteristics, as our study focused on vulnerable women receiving cash transfers, including household heads, undernourished individuals, breastfeeding, and pregnant women. Variations in wealth levels, geographic locations, and analytical methods may contributed to the observed.

### Food consumption score among non-beneficiaries in context of other studies

Among non-cash beneficiaries’ women, more than half (55.9%) were in the borderline category, and nearly one-quarter (23.3%) were in the poor category, showing more acute difficulty in achieving dietary needs. Only 20.8% achieved an acceptable FCS. The acceptable food consumption score is much lower than the study conducted in. rural South Ethiopia [24]. afar and Somali region [49]. Konso Zone, Southwestern Ethiopia [14] and Owo Township, Ondo State, Nigeria [43] study conducted in five woredas of East Gojjam Zone, Amhara, Ethiopia [44]. Jashore district in Bangladesh [50] in which shows 73.9%,38.75%, 81.5%,56.1%, 80.3%, 58%, respectively. This gap may be caused by inadequate food availability, inadequate food usage, improper dietary practices, variations in the research population and study participants.

Our finding is still far below than National Food Consumption Score Vulnerability Assessment and Mapping report that the level of food consumption in Ethiopia and WFP recommendation which is 74% [46] and 90% [47]. The study revealed that the borderline FCS was 55.9%, and poor FCS was 23.3% This study significantly higher compared to previous studies conducted in Ethiopia in Konso Zone, southern Ethiopia (17.4%) borderline and (14.1%) poor FCS [14]. Amara (11%), Tigray (16%), Afar (6%), and Somali (17%), and 1.9% poor and 16% border line of FCS in Shegaw Motta Hospital, East Gojjam Zone, Ethiopia [15]. This research focused on women receiving cash transfers who are particularly vulnerable, including heads of households and undernourished individuals, as well as nursing and pregnant women. The differences observed may be attributed to variations in the study environment and the characteristics of the participants. Additionally, the disparities noted could have been affected by variations in methodological approaches, geographic context, and socioeconomic status.

### Factors associated with food consumption score among beneficiary

As age increases, there is a negative correlation with food consumption scores among beneficiaries. When comparing mothers aged 18–24 to those in the 25–34, 35–44, and 45–49 age brackets, the likelihood of achieving an acceptable Food Consumption Score (FCS) decreases by 72%, 77%, and 89%, respectively. This finding is consistent with research conducted in Addis Ababa [51]. It is plausible that younger women enjoy better access to food and a more diverse diet, whereas older women experience reduced dietary diversity due to financial constraints and household responsibilities.

Larger families were significantly less likely to attain an acceptable Food Consumption Score (FCS) when compared to smaller families. The likelihood of achieving a superior FCS in larger households is 87% lower than that in smaller households. This observation is consistent with research conducted in Addis Ababa [16]. This may be attributed to the fact that as family size increases, resources such as food may become more limited, thereby diminishing dietary diversity.

Beneficiaries residing in households that include a member with special needs were 58% less likely to attain an acceptable Food Consumption Score (FCS) in comparison to households that do not have members with special needs. This observation is consistent with research conducted in Hawassa [52]. The disparity may be attributed to the increased financial and caregiving responsibilities associated with special needs, which diminishes available resources.

Households that faced conflicts regarding the utilization of cash support were 83% less likely to attain an acceptable Food Consumption Score (FCS) in comparison to households that did not experience such conflicts. This observation is consistent with research conducted in Hareri [28]. The underlying reason may be that disagreements or disputes concerning the application of cash assistance can hinder the effective distribution of resources for food, consequently diminishing dietary diversity and overall food security within the household.

Mothers with good dietary habits were twice as likely to achieve an acceptable (FCS) compared to those with poor dietary habits this finding aligns with studies conducted in Gondar [53]. This may involve incorporating a variety of food groups and organizing meal plans.

Unexpectedly, women who knew a lot about nutrition were less likely to have a higher Food Consumption Score. This may suggest that when households deal with financial limitations, rising food prices, and restricted access to a variety of foods, knowledge alone does not ensure better eating habits. Furthermore, this link might have been impacted by residual confounding given the cross-sectional design. To investigate this relationship, more long-term research is required [28].

### Factors associated with food consumption score among non-beneficiary

Households with middle wealth had almost double the likelihood of attaining a higher Food Consumption Score (FCS) in comparison to impoverished households, potentially attributable to improved access to a variety of foods and enhanced financial resources, as supported by research conducted in Bahir Dar [54]. Although the Urban Productive Safety Net Programme is designed to assist economically disadvantaged households, a significant number of participants were identified as belonging to middle and affluent categories. This situation likely indicates the application of a relative wealth index (utilizing Principal Component Analysis) based on urban household assets, which may fail to adequately account for income volatility and food insecurity—essential factors for determining eligibility for the program.

A larger family size has a detrimental impact on Food Consumption Score (FCS), as households with a greater number of members are 96.1% less likely to attain a higher score. This phenomenon may be attributed to the sharing of food among more individuals, which diminishes per capita dietary diversity, corroborating the results observed in Dire Dawa [55].

The existence of a household member with special needs decreased the likelihood of achieving an acceptable Food Consumption Score (FCS) by 46%, which aligns with findings from southern Ethiopia [56].This reduction may be attributed to the extra caregiving responsibilities and financial strains that restrict the resources allocated for food.

Favorable attitudes regarding nutrition enhanced the probability of attaining elevated Food Consumption Scores (FCS), and mothers possessing sound nutritional knowledge were notably more inclined to achieve superior scores, aligning with findings from Addis Ababa [53]. This may be attributed to a greater awareness of healthy dietary practices.

Good dietary practices were associated with higher FCS, with women reporting healthier habits having 24% higher odds of achieving a better score corroborating studies from Bangladesh [57]. May be due to regular consumption of diverse and nutrient-rich foods.

Good nutrition knowledge was positively associated with higher food consumption scores, with women who had good nutrition knowledge having 73% higher odds of achieving a better food consumption score [58].

## Strength and limitation

To ensure the accuracy of food consumption data, this study utilized a validated food frequency questionnaire adapted from the United Nations High Commissioner for Refugees (UNHCR) and the World Food Programme (WFP) (UNHCR, 2021) [59]. The relevance of the food items assessed was enhanced through the involvement of local key informants knowledgeable about the language, culture, and dietary practices. Although the Food Consumption Score (FCS) and food security are often considered related, these recommendations are not the only means to improve food security. However, the cross-sectional study design limits the ability to establish causal relationships between outcome and predictor variables. While the FCS is a standardized method for evaluating overall dietary adequacy, it does not capture the intake of specific macronutrients and micronutrients. Data collection during a single season may also limit the findings’ applicability to other times of the year. Additionally, response bias may have arisen from cash beneficiaries’ expectations of benefit-related incentives, and recall bias could have occurred due to participants relying on a seven-day memory period.

## Conclusion

This study examined the Food Consumption Score (FCS) and its determinants among women in Gondar City, comparing cash beneficiaries with non-beneficiaries. Among beneficiaries, 50.0% achieved an acceptable Food Consumption Score compared with 20.8% of non-beneficiaries. Falling short of the standards established by the World Food Programme (WFP) and national guidelines. Financial support and adherence to healthy eating practices emerged as significant predictors of FCS; however, increasing age negatively affected FCS. Among beneficiaries, larger family sizes, the presence of individuals with special needs, and household conflicts were associated with lower FCS, while healthy eating practices had a positive impact. For non-beneficiaries, larger families and members with special needs also correlated with reduced FCS, whereas factors such as middle-class wealth, positive attitudes toward nutrition, healthy eating behaviors, and nutritional knowledge were linked to improved FCS.

Policymakers should strengthen and expand urban safety-net interventions while integrating nutrition education, livelihood support, and income-generating opportunities to improve household dietary diversity and food security among vulnerable women.

## Data Availability

The data used for this study will be available from corresponding author upon reasonable request.

## Abbreviations

ANRS: Amhara National Regional State
CI: Confidence Interval
EDHS: Ethiopia Demographic and Health Survey
FCSs: Food Consumption Scores
GHI: Global Hunger Index
HHs: Households
IRPBH: Institutional Review Board of institute of Public Health
LMIC: - Low and Middle-Income Countries
NGO: Non-Governmental Organizations
PLW: Pregnant and Lactating Women
SSA: Sub-Saharan African
UPSNP: Urban Productive Safety Net Program
UNCHR: United Nations High Commissioner for Refugees
WFP: World Food Programs
WHO: World Health Organization
